# Prediction of total and regional body composition from 3D body shape

**DOI:** 10.1038/s41746-024-01289-0

**Published:** 2024-10-23

**Authors:** Chexuan Qiao, Emanuella De Lucia Rolfe, Ethan Mak, Akash Sengupta, Richard Powell, Laura P. E. Watson, Steven B. Heymsfield, John A. Shepherd, Nicholas Wareham, Soren Brage, Roberto Cipolla

**Affiliations:** 1 Department of Engineering, University of Cambridge, Cambridge, UK; 2 MRC Epidemiology Unit, Cambridge Biomedical Campus, Hills Road, Cambridge, CB2 OQQ UK; 3 NIHR Cambridge Clinical Research Facility, Cambridge University Hospitals, Cambridge, UK; 4 Metabolism & Body Composition Laboratory, Pennington Biomedical Research Center, Baton Rouge, LA USA; 5 Population Sciences in the Pacific Program (Cancer Epidemiology), University of Hawaii Cancer Center, Honolulu, HI USA

**Keywords:** Epidemiology, Obesity

## Abstract

Accurate assessment of body composition is essential for evaluating the risk of chronic disease. 3D body shape, obtainable using smartphones, correlates strongly with body composition. We present a novel method that fits a 3D body mesh to a dual-energy X-ray absorptiometry (DXA) silhouette (emulating a single photograph) paired with anthropometric traits, and apply it to the multi-phase Fenland study comprising 12,435 adults. Using baseline data, we derive models predicting total and regional body composition metrics from these meshes. In Fenland follow-up data, all metrics were predicted with high correlations (*r* > 0.86). We also evaluate a smartphone app which reconstructs a 3D mesh from phone images to predict body composition metrics; this analysis also showed strong correlations (*r* > 0.84) for all metrics. The 3D body shape approach is a valid alternative to medical imaging that could offer accessible health parameters for monitoring the efficacy of lifestyle intervention programmes.

## Introduction

Body composition is strongly related to the risk of chronic disease morbidity and mortality^1^, and can be assessed accurately using medical imaging methods such as dual-energy X-ray absorptiometry (DXA), magnetic resonance imaging (MRI) and computed tomography (CT)^2–4^. However, these methods are not readily available to be used routinely in clinical practice and in epidemiological studies due to practical and ethical constraints, nor are they easily accessible to the general public^5,6^. In these settings, conventional anthropometry such as body mass index (BMI), waist, hip circumferences and waist-hip ratio are typically used to infer body composition. Parente et al.^7^ determine waist-height ratio and waist as best estimators for visceral fat in type-1 diabetes. Heymsfield et al.^8^ analyse simple skeletal muscle mass prediction formulas in different ethnicities. However, these indirect methods of assessing body composition are insufficiently accurate or convenient for longitudinal use as they often require face-to-face clinical visits and trained staff. Furthermore, these surrogate measures do not differentiate between fat and lean mass or their distribution^9,10^.

There is a need to develop simple, accessible, and relatively inexpensive tools to improve the accuracy of assessing body composition. This would provide better prediction of metabolic health and identify people at high risk of disease in the long term so that remedial action can be taken. Significant works in recent years have focused on the development of 3D optical (3DO) scanning^11^ to estimate body composition^12–15^. 3DO scanners use depth sensors by projecting infrared patterns onto the scan subject to rapidly construct a 3D point cloud using multiview stereo, and subsequently capture 3D surface shape information. Rather than predicting body composition from anthropometric measurements alone, 3D body shape as a whole provides more visual and implicit cues for predicting body composition more accurately. Additional 3D shape cues can either be additional landmark diameters, circumferences, surface areas and volumes from 3DO scans^16^, or parameters of a PCA shape space^13–15^. More recently Leong et al.^17^ use a variational autoencoder (VAE)^18^ to learn latent DXA encoding, and map 3DO scans to pseudo-DXA images. These works have shown that 3D shape information could augment conventional prediction models using anthropometry, or outperform them as a standalone predictor for a variety of body composition metrics. However, while the cost of 3DO scanners is comparatively lower than that of DXA, MRI or CT, obtaining 3DO scans still requires a dedicated apparatus, which makes it less accessible to the general public.

To derive shape information without full reliance on 3DO scanners to reconstruct 3D body meshes, recent works have taken advantage of developments in computer vision and machine learning algorithms, which have enabled accurate segmentation^19^ and pose estimation^20^ of objects including the human body from RGB images, which are easily obtainable using a smartphone camera. Majmudar et al.^21^ train a convolutional neural network (CNN) to directly predict percentage body fat from front and back images. Alves et al.^22^ locate key points from multiple views, derive circumferences, and predict percentage body fat. Xie et al.^23^ construct a PCA shape space from 2D DXA silhouettes and predict body composition. Sullivan et al.^24^ derive body volume from a single image by measuring horizontal landmark diameters and use the volume in a 3-compartment model (body mass, body volume and body water) to calculate percentage body fat. Smith et al.^25^ compare circumference estimation accuracy using a smartphone app and 3DO scanners, and claim that circumferences estimated from images can be relatively accurate. McCarthy et al.^16^ derive lengths, circumferences and volumes from body shape images, and predict skeletal muscle mass from these measurements alongside demographic variables. Graybeal et al.^26^ evaluate two smartphone apps and compare circumference and circumference ratio prediction accuracy. The remote data capture and modelling of 3D shapes has numerous applications, including helping patients track individual changes over time for commonly assessed anthropometric measurements. Furthermore, patients are not required to physically attend clinics to have these measures done, thus lowering the burden on health services and providing a more cost-effective way to monitor aspects of patient health.

Unfortunately, there are few large-scale datasets containing 3D body meshes with paired anthropometric and metabolic traits. Bennett et al.^12^ worked with a cohort of size 501, McCarthy et al.^16^ had a cohort size of 322, and Ng et al.^14^ had a cohort size of 407. These small cohorts prevent larger deep-learning models from being leveraged for body composition predictions. Klarqvist et al.^27^ used a large-scale MRI database from the UK Biobank^28^, to predict body composition from coronal and sagittal silhouettes. To the best of our knowledge, this is the only study that contains MRI data at this scale, as the use of MRI in population studies is limited due to cost and accessibility for research. 3D body shape datasets are therefore scarce, while datasets containing 2D DXA images with anthropometric and metabolic traits (body composition) are in abundance.

Therefore in this work, we present a novel method that first fits 3D body meshes to DXA silhouettes and paired anthropometry measurements consisting of height, waist and hip circumferences. Similar to our approach, Keller et al.^29^ register a 3D mesh to a DXA silhouette to infer skeletal structure, and Tian et al.^30^ fit a 3D mesh to a pose-constrained frontal image. However, these methods are limited to the coronal silhouette, while our method injects sagittal information in the form of waist and hip circumferences. Using our method, we generate a large 3D body shape database of 17,461 meshes. We then show that using the fitted meshes, total and regional body composition metrics can be predicted accurately.

We also test and evaluate the performance of a smartphone app (3D Body Shape App) that uses phone images alone to make it easier for individuals to visualise and track changes in their body shape^31^. The app captures four photographs (front, back, left-side, and right-side profiles of the participant), and reconstructs a 3D body mesh using these images. McCarthy et al.^16^ and Smith et al.^25^ also generate 3D body meshes from RGB images using an app, which is the most similar to our smartphone approach. However, their app requires constrained A-pose for the photographs and could fail due to noisy background in our testing. In contrast, our method is robust to background, participant poses, camera orientation, and could be extended to accept an arbitrary number of input images. We show preliminary body composition prediction results using the app. Our aim is to improve performance by increasing reconstruction accuracy in the future.

In summary, we make the following contributions in this study:

- Construct a large 3D body shape database derived from 2D DXA silhouettes and paired anthropometry measurements (height, waist and hip circumferences);
- Predict, from 3D body shape, total and regional body composition metrics including:
  — Total fat mass;
  — Percentage body fat (PBF);
  — Android fat mass;
  — Gynoid fat mass;
  — Peripheral fat mass;
  — Visceral adipose tissue (VAT) mass;
  — Abdominal subcutaneous adipose tissue (SCAT) mass;
  — Total lean mass;
  — Appendicular lean mass;
  — Appendicular lean mass index (ALMI).
- Evaluate and show preliminary results of a smartphone app that predicts body composition by reconstructing 3D body meshes from images only.

To the best of our knowledge, our method is the first that fits 3D body meshes to DXA images and predicts downstream body composition metrics. In this way, we show that accurate 3D meshes can be derived from a single 2D silhouette plus simple anthropometry (height, waist and hip circumferences), from which body composition metrics can be predicted.

## Results

The demographic and anthropometric characteristics of the Fenland study samples and the smartphone validation study are summarised in Table 1. Participants in the smartphone validation study were younger, lighter and leaner, compared to participants in the Fenland study. In terms of body volume, we observed a mean (SD) of 64.4 (13.2) liters in the smartphone validation study, the only dataset with air plethysmography measures.

**Table 1:** Participant characteristics

Variables	units	Fenland Study			Smartphone validation study
		Phase 1 (training)	Phase 1 (validation)	Phase 2 (validation)	
		*n* = 9087	*n* = 2272	*n* = 6102	*n* = 119
Age	yrs	48.7 ± 7.5	48.8 ± 7.6	55.8 ± 7.05	42.3 ± 12.3
Weight	kg	77.6 ± 15.3	77.6 ± 15.3	77.3 ± 15.2	69.9 ± 13.5
Height	cm	170.0 ± 9.3	170.0 ± 9.3	170.4 ± 9.3	168.4 ± 9.8
BMI	kg/m^2^	26.8 ± 4.5	26.7 ± 4.5	26.6 ± 4.4	24.6 ± 4.2
Sex	*n* (%) male	4298 (47.3)	1035 (45.6)	2979 (48.8)	39 (32.7)
Total body fat mass	kg	26.4 ± 9.0	26.5 ± 9.2	26.3 ± 9.0	21.0 ± 9.0
Percentage body fat	%	33.5 ± 7.7	33.8 ± 8.0	33.7 ± 7.8	29.4 ± 9.5
Android fat mass	kg	2.3 ± 1.2	2.3 ± 1.2	2.4 ± 1.2	1.5 ± 1.1
Gynoid fat mass	kg	4.4 ± 1.6	4.4 ± 1.6	4.3 ± 1.5	3.9 ± 1.5
Visceral fat mass	kg	1.0 ± 0.8	1.0 ± 0.8	1.0 ± 0.8	0.5 ± 0.5
Abdominal SCAT mass^a^	kg	1.4 ± 0.6	1.4 ± 0.7	1.3 ± 0.6	1.1 ± 0.8
Peripheral fat mass^b^	kg	11.6 ± 4.0	11.7 ± 4.1	11.3 ± 4.0	10.2 ± 4.0
Total lean mass	kg	48.8 ± 10	48.4 ± 9.8	48.2 ± 10	46.8 ± 10
Appendicular lean mass^c^	kg	22.3 ± 5.5	22.1 ± 5.4	21.6 ± 5.3	21.7 ± 5.5
ALMI^d^	kg/m^2^	7.6 ± 1.3	7.5 ± 1.3	7.4 ± 1.3	7.5 ± 1.3

Data are mean ± SD.

^a^SCAT = subcutaneous adipose tissue.

^b^Peripheral fat mass = arms + legs fat mass.

^c^Appendicular lean mass = arms + legs lean mass.

^d^ALMI: appendicular lean mass index = appendicular lean mass/height^2^.

### 3D body mesh fitting

**Fig. 1:**
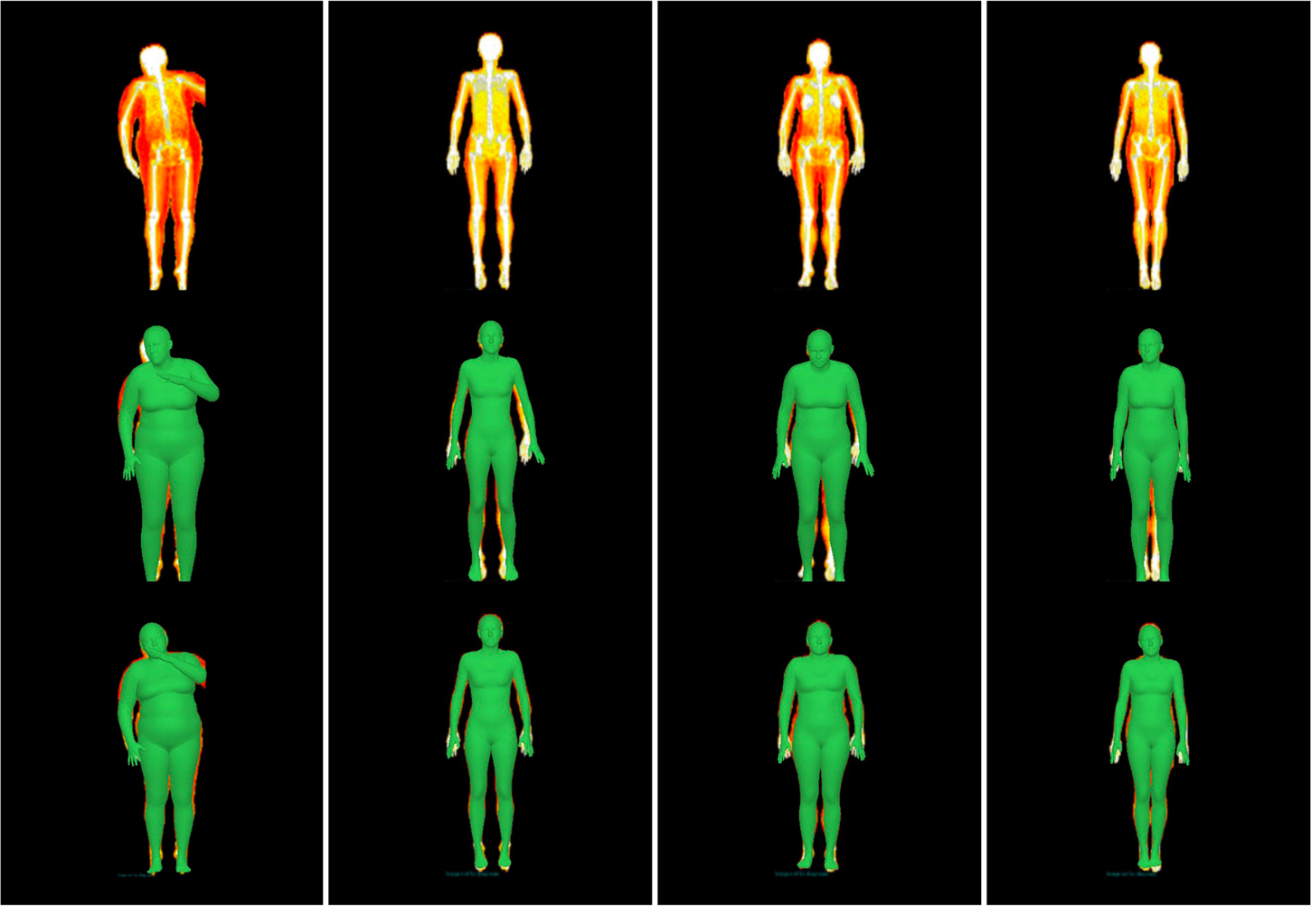
Body meshes fitted to DXA. DXA image inputs (Row 1), initial fits using HKPD (Row 2) and optimised fits (Row 3). Initial fits are not accurate enough to represent the participant. Optimised fits have better agreements with the DXA silhouettes.

Figure 1 shows samples of our fitted 3D body meshes. We show individuals from different weight groups to qualitatively demonstrate that our fitting pipeline works for different body shapes. Row 1 shows the raw DXA scans. Row 2 shows the initial pose and shape estimations obtained using HKPD^20^. These roughly capture the pose and shape of the body, but the fit to the coronal silhouette is often poor. Row 3 of Fig. 1 shows samples of optimised fits. We found that optimised meshes agree much better with the silhouettes of DXA images compared with the initial fit. Furthermore, Supplementary Fig. 1 shows samples of meshes before and after optimisation in sagittal view. We observed that the optimisation has resulted in significant changes to the body shapes in the depth dimension when comparing the initial meshes to the optimised meshes. This further shows that initial fits do not represent the actual body shape and that waist and hip circumferences were needed to generate meshes that better represent the true body shape of participants. Our method, in conclusion, has generated body meshes that are injected with 3D body shape information using paired anthropometry, while creating an improved fit to the DXA silhouettes. SMPL shape parameters corresponding to these optimised meshes are then used for the body composition regressor.

### Body composition prediction

**Fig. 2:**
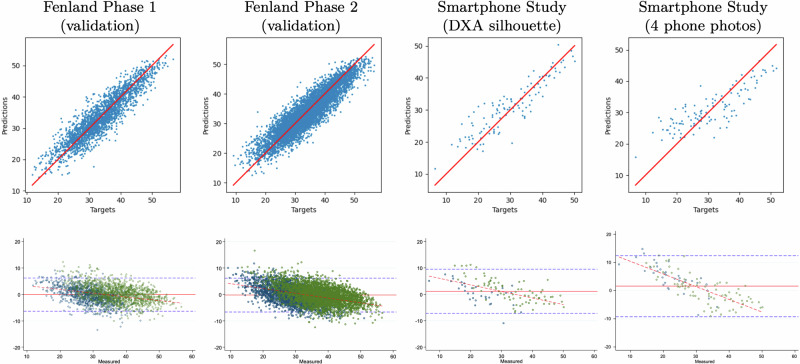
Body fat prediction scatter plots and Bland–Altman plots. Scatter plots (Row 1) and Bland–Altman plots (Row 2) for agreement between predicted and measured percentage body fat in the Fenland samples and the external validation set. All predictions are based on 3D meshes derived from DXA silhouettes, except last panel which is based on 3D meshes derived from four RGB photos from smartphone.

Table 2 shows the model performance on Fenland and smartphone datasets in the form of mean bias (95% limits of agreement), root-mean-square error (RMSE), and Pearson correlation for the agreement between ground truth body composition values from DXA and model predictions using the 3D meshes. The last column in this table is the model performance using the smartphone method. Supplementary Fig. 2 shows selected scatter plots of model predictions against target values on Fenland phase 2 data. Figure 2 shows scatter plots and Bland–Altman plots for percentage body fat for Fenland phase 1 (validation), phase 2 (validation), smartphone study using DXA silhouette optimisation, and smartphone study using RGB images.

**Table 2:** Agreement analysis of 3D body shape predicted vs measured body composition

		Fenland phase 1 (validation)			Fenland phase 2 (validation)			Smartphone Validation Study					
		DXA silhouette			DXA silhouette			DXA silhouette			4 phone photos		
		*n* = 2272			*n* = 6102			*n* = 119			*n* = 119		
		Bias (95%LoA)	RMSE	*r*^a^	Bias (95%LoA)	RMSE	*r*^a^	Bias (95%LoA)	RMSE	*r*^a^	Bias (95%LoA)	RMSE	*r*^a^
DXA	Total body fat mass (kg)	−0.09 (−4.9; 4.7)	2.45	0.96	-0.13 (-5; 4.8)	2.50	0.96	0.57 (-5; 6.2)	2.86	0.95	0.93 (-6.4; 8.3)	3.76	0.91
	Percentage body fat (%)	−0.07 (−6.4; 6.2)	3.22	0.91	−0.24 (−6.7; 6.2)	3.28	0.91	1.13 (−7.2; 9.5)	4.26	0.90	1.62 (−9.2; 12.5)	5.54	0.84
	Android fat mass (kg)	0 (−0.7; 0.8)	0.38	0.95	0.03 (−0.7; 0.8)	0.38	0.95	0.07 (−0.6; 0.8)	0.36	0.95	0.12 (−0.9; 1.1)	0.50	0.89
	Gynoid fat mass (kg)	0.04 (−1; 1)	0.51	0.95	0.02 (−1; 1)	0.51	0.94	0.06 (−1.1; 1.2)	0.59	0.92	0.13 (−1.2; 1.3)	0.67	0.90
	Visceral fat mass (kg)	−0.03 (−0.7; 0.6)	0.34	0.89	−0.04 (−0.8; 0.7)	0.37	0.90	−0.02 (−0.4; 0.4)	0.19	0.94	0.08 (−0.5; 0.7)	0.29	0.85
	Abdominal SCAT mass (kg)^b^	0.05 (−0.5; 0.6)	0.30	0.90	0.09 (−0.5; 0.7)	0.31	0.86	0.07 (−0.7; 0.9)	0.42	0.89	0.03 (−0.9; 1.0)	0.49	0.86
	Peripheral fat mass (kg)^c^	−0.06 (−2.7; 2.6)	1.36	0.94	−0.11 (−2.8; 2.6)	1.39	0.93	0.12 (−2.6; 2.9)	1.40	0.94	0.44 (−3.0; 3.9)	1.74	0.91
	Total lean mass (kg)	0.11 (−4.7; 4.9)	2.45	0.97	0.32 (−4.7; 5.3)	2.54	0.97	−0.95 (−6.9; 5)	3.05	0.95	−1.51 (−9.0; 6.0)	3.82	0.93
	Appendicular lean mass (kg)^d^	−0.01 (−2.8; 2.7)	1.40	0.97	0.41 (−2.3; 3.2)	1.40	0.97	−1.21 (−4.2; 1.8)	1.53	0.96	−1.25 (−5.3; 2.8)	2.05	0.93
	ALMI (kg/m^2^)^e^	−0.01 (−0.9; 0.9)	0.48	0.93	0.14 (−0.8; 1.1)	0.48	0.93	−0.43 (−1.5; 0.6)	0.54	0.91	−0.42 (−1.8; 1.0)	0.70	0.84
BODPOD	Volume (L)	–	–	–	–	–	–	–	–	–	5.36 (−4.9; 15.6)	5.21	0.91
Anthropometry	Waist Circumference (cm)	–	–	–	–	–	–	–	–	–	2.49 (−7.4; 12.4)	5.04	0.89
	Hip circumference (cm)	–	–	–	–	–	–	–	–	–	2.25 (−6.3; 10.8)	4.38	0.87
	Calf circumference (cm)	–	–	–	–	–	–	–	–	–	4.09 (1.3; 6.7)	2.76	0.59
	Arm circumference (cm)	–	–	–	–	–	–	–	–	–	−3.03 (−7.8; 1.7)	2.42	0.80

^a^*r* = Pearson’s correlations.

^b^SCAT = subcutaneous adipose tissue.

^c^Peripheral fat mass = arms + legs fat mass.

^d^Appendicular lean mass = arms + legs lean mass.

^e^ALMI: appendicular lean mass index = appendicular lean mass/height^2^.

In the Fenland phase 1 validation sample, correlation coefficients between predicted and measured DXA parameters were strong (*r* > 0.89) for all fat mass and lean mass variables. Bland–Altman analyses revealed no significant mean bias for the following predicted DXA parameters: total fat mass, percentage body fat, android fat, appendicular lean mass and appendicular lean mass index (all *P* > 0.05). However, significant (*P* < 0.05) mean bias was observed for gynoid fat, visceral fat, abdominal SCAT mass, peripheral fat and total lean mass. In the Fenland phase 2 (validation) sample, which included now older individuals, correlation coefficients between predicted and measured DXA parameters were also strong (*r* > 0.86) for all the body composition variables. Agreement analyses revealed significant (*P* < 0.05) mean bias for all predicted DXA measured parameters. Mean bias was −0.24 (−6.7; 6.2)% for percentage body fat and for the other body composition metrics, the mean bias ranged between -0.04 to 0.41 kg. Similar results for the DXA silhouette method were also observed in the external validation (Column 3 of Table 2), which included younger individuals. Correlation coefficients between predicted and measured DXA parameters were *r* > 0.89. Mean bias was 1.13 (−7.2; 9.5)% for percentage body fat and for the other body composition parameters, the mean bias ranged between −1.21 to 1.13 kg.

Results using SMPL shape generated directly from the four smartphone images in this external validation are shown in Column 4 of Table 2. The correlation coefficients between DXA metrics and all the predicted body composition values were *r* > 0.84. Mean bias (95% LoA) was 1.62 (−9.2; 12.5)% for percentage body fat and for the other body composition parameters, the mean bias ranged between −1.51 to 0.93 kg. Volume derived from the smartphone achieved RMSE of 5.21 L compared to BODPOD volume, with mean bias (95% LoA) of 5.36 (−4.9; 15.6) L. We also compared accuracy of waist, hip, calf, and arm circumferences from the smartphone avatars, which achieved RMSE of 5.04 cm, 4.38 cm, 2.76 cm and 2.42 cm, respectively.

### Comparison between prediction models

We conducted a comparison study on different regressor model inputs, to verify that 3D body meshes provide crucial information for the downstream composition regressor. Results of the different models investigated in the comparison study are shown in Table 3. Model A (weight and height only) achieved some level of predictive ability. Performance of the model was improved by adding waist and hip circumferences (Models B and C), as waist and hip are strong indicators of composition metrics such as android fat mass and gynoid fat mass. In the final model (Model E), we quantified the contribution of the SMPL shape parameters in addition to using height and weight only. This model substantially improved the estimation of the body composition metrics compared to anthropometry alone. The explained variance (*R*^2^) in percentage body fat increased from 73% to 82%; total fat mass from 88% to 92%; total lean mass from 91% to 93%; gynoid fat from 81% to 89%; android fat from 81% to 89%; peripheral fat mass from 80% to 87%; appendicular lean mass 90 to 93%; appendicular lean mass index from 74% to 86%; visceral fat from 70% to 80% and abdominal SCAT from 70% to 72%. We also attempted to predict body composition using a simple linear regressor (Model D), but the neural network approach (Model E) outperformed it noticeably.

**Table 3:** Comparison study on model inputs

Model Name	A	B	C	D	E
Model Inputs	–	–	–	SMPL	SMPL
	Height, Weight	Height, Weight	Height, Weight	Height, Weight	Height, Weight
	–	Waist	Waist, Hip	–	–
Method	Network	Network	Network	Linear	Network
Metrics	Fenland phase 2 *R*^2^				
Total fat mass	0.884	0.909	0.910	0.897	**0.922**
Percentage body fat	0.739	0.792	0.797	0.766	**0.823**
Android fat mass	0.809	0.887	0.884	0.880	**0.894**
Gynoid fat mass	0.811	0.830	0.863	0.843	**0.886**
Visceral fat mass	0.698	0.779	0.792	0.774	**0.802**
Abdominal SCAT mass^a^	0.701	0.727	**0.730**	0.716	0.723
Peripheral fat mass^b^	0.802	0.824	0.832	0.821	**0.872**
Total lean mass	0.910	0.925	0.921	0.916	**0.934**
Appendicular lean mass^c^	0.895	0.921	0.911	0.906	**0.927**
ALMI^d^	0.739	0.853	0.824	0.833	**0.853**

Best *R*-squared values are in bold font.

^*a*^SCAT = subcutaneous adipose tissue.

^*b*^Peripheral fat mass = arms + legs fat mass.

^*c*^Appendicular lean mass = arms + legs lean mass.

^*d*^ALMI: appendicular lean mass index = appendicular lean mass/height^2^.

### Predictions of body composition change

A total of 5733 individuals participated in both Fenland Phase 1 and Phase 2, which enabled us to examine the model’s ability to detect within-individual body composition changes over a mean (SD) of 6.7 (2.0) years.

**Fig. 3:**
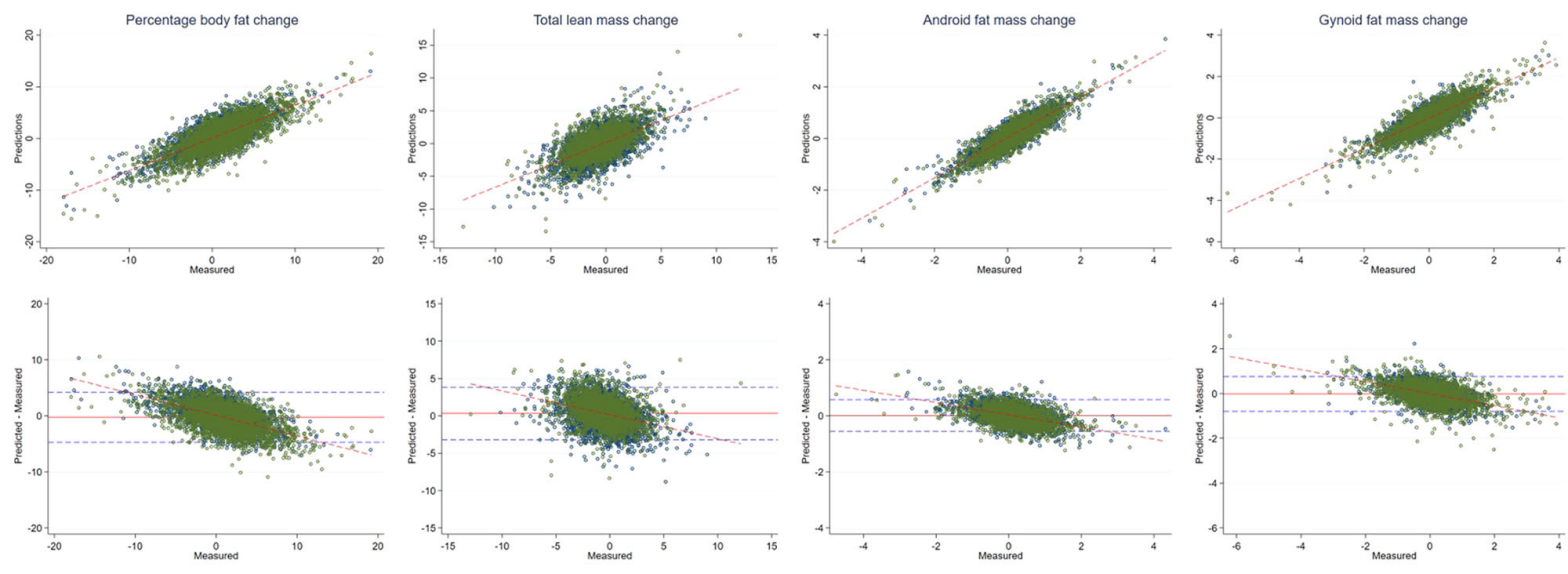
Prediction scatter plots of body composition changes. Selected scatter plots (Row 1) and corresponding Bland–Altman plots (Row 2) for prediction of change in body composition metrics.

Table 4 shows model predictions of changes in body composition metrics for individuals present in both Fenland phases. Our model was able to detect change for numerous fat mass metrics. The agreement between predicted body composition values and DXA parameters (*r*) for changes in percentage body fat, total fat mass, gynoid fat mass, android fat mass, peripheral fat mass, visceral fat mass and abdominal SCAT mass were 0.92, 0.76, 0.87, 0.83, 0.74, 0.76, 0.82, respectively, with RMSE of 2.3%, 1.75 kg, 0.39 kg, 0.29 kg, 1.00 kg, 0.26 kg, 0.21 kg respectively. Changes in lean mass were less well captured, mainly due to the fact that lean mass largely remains unchanged for most individuals over this time period. *r* values for change for total lean mass, appendicular lean mass and ALMI were 0.60, 0.64, and 0.63, respectively, with RMSE of 1.82 kg, 1.06 kg, and 0.36 kg respectively. Figure 3 shows selected scatter plots between the predicted changes in percentage body fat, lean mass, android and gynoid fat mass against changes measured by DXA for the same variables and the corresponding Bland–Altman plots.

**Table 4:** Predictions of body composition changes for participants in both Fenland phases

Metrics	Bias (95%LoA)	RMSE	*r*
Total fat mass	−0.11 (−3.5; 3.3)	1.75	0.92
Percentage body fat	−0.25 (−4.7; 4.2)	2.30	0.76
Android fat mass	0.01 (−0.6; 0.6)	0.29	0.87
Gynoid fat mass	−0.02 (−0.8; 0.7)	0.39	0.83
Visceral fat mass	−0.02 (−0.5; 0.5)	0.26	0.74
Abdominal SCAT mass^a^	0.03 (−0.4; 0.4)	0.21	0.76
Peripheral fat mass^b^	−0.05 (−2.0; 1.9)	1.00	0.82
Total lean mass	0.32 (−3.2; 3.8)	1.82	0.60
Appendicular lean mass^c^	0.45 (−1.4; 2.3)	1.06	0.64
ALMI^d^	0.16 (−0.5; 0.8)	0.36	0.63

^a^SCAT = subcutaneous adipose tissue.

^b^Peripheral fat mass = arms + legs fat mass.

^c^Appendicular lean mass = arms + legs lean mass.

^d^ALMI: appendicular lean mass index = appendicular lean mass/height^2^.

## Discussion

In this paper, we derived a novel computer vision-based method that fits a 3D body mesh to a single DXA silhouette with paired anthropometry data (height, waist and hip circumferences). Using our method, we generated a large database of 3D body meshes (*n* = 17,461) with paired anthropometric and metabolic traits. We then showed that total and regional body composition metrics could be predicted accurately using these meshes. In the comparison study, we showed that shape parameters provide additional cues for predicting body composition by comparing the performance of models with different inputs. We demonstrated the derived model’s ability to detect longitudinal change in these characteristics over time by making predictions for individuals that were present in both phases of the Fenland study. In our smartphone validation study, we showed how avatars were generated using four smartphone photographs directly, without the use of optimisation. Finally, we showed preliminary body composition prediction results using these avatars.

The model using optimised 3D meshes predicted body composition metrics with sufficient accuracy to assess relative differences between individuals and was sufficiently accurate to predict absolute values for total and regional body composition including visceral fat and abdominal SCAT mass, as well as lean mass, appendicular lean mass, and ALMI. All the body composition metrics predicted from the optimised 3D meshes had small significant mean bias and showed Pearson correlation coefficients *r* > 0.86. These mean biases were likely caused by the older population in the phase 2 cohort compared to phase 1 used for training. Nevertheless, the mean biases were small (less than 2% for all metrics except visceral and abdominal SCAT mass (4%, and 7% respectively)), and the corresponding scatter plots showed strong prediction results (Fig. 2, Supplementary Fig. 2). In addition, our prediction model achieved similar performance for different body groups when stratified by BMI and sex (Supplementary Table 1). Our model outperformed those using traditional anthropometry^7,8^ discussed previously as expected. In a similar study, Xie et al.^23^ which used a 2D PCA shape space constructed from key points selected on a silhouette achieved the following results on a cohort of size 1554: percentage body fat *R*^2^ adj. (adjusted *R*^2^) = 0.728, RMSE = 3.12% for boys, and *R*^2^ adj. = 0.691, RMSE = 3.39% for girls. However, this comparison is limited as this study was conducted in children, as they have proportionally larger body surface area to volume ratio than adults, as well as sex differences in dimensions of body shapes such as central:peripheral ratio^32^. In a more relevant study, Ng et al.^14^ which predicted composition using PCA parameters of 3DO scans on a cohort of size 407, observed similar results to our analysis: total fat mass *R*^2^ = 0.91, RMSE = 3.07 kg for males, *R*^2^ = 0.95, RMSE = 2.63 kg for females; percentage body fat *R*^2^ = 0.70, RMSE = 3.55% for males, *R*^2^ = 0.72, RMSE = 3.88% for females. Similarly, Tian et al.^30^ fitted a 3D shape to a single coronal silhouette on a cohort of size 416, and predicted body composition with the following results: total fat mass *R*^2^ = 0.90, RMSE = 3.63 kg for males, *R*^2^ = 0.94, RMSE = 2.29 kg for females; percentage body fat: *R*^2^ = 0.725, RMSE = 3.90% for male, *R*^2^ = 0.74, RMSE = 3.29% for female. In comparison, we achieved similar performance on a much larger dataset *n* = 6102, with the following results: total fat mass *R*^2^ = 0.922, RMSE = 2.5 kg; percentage body fat *R*^2^ = 0.823, RMSE = 3.28% overall. In addition, our approach also injects information in the depth dimension by using waist and hip circumferences, while the fitting by Tian et al.^30^ is limited to the coronal silhouette. In Klarqvist et al.^27^, stronger correlations were observed for visceral fat, abdominal SCAT mass and gynoid fat, using coronal and sagittal silhouettes derived from MRI, the gold standard for those measures of adiposity. Our estimates for these metrics were based on an in-built algorithm from the DXA manufacturer, which is not a criterion method. However, compared to Klarqvist et al.^27^, our analysis included more body composition metrics such as appendicular lean mass and its index, which are used as a proxy for the assessment of sarcopenia^33,34^. Even though comparison to these studies may be limited as they were conducted in cohorts of different ages, using different body composition instruments and computer vision approaches, we have shown that our method produced meshes that were comparably accurate to 3DO scans^14^, by predicting body composition to a similar accuracy on a large test dataset. This allows for the construction of large 3D shape databases using our method, and in turn, enables larger deep-learning models to be used to analyse 3D body shapes. Our prediction results for change in body composition were similar to Wong et al.^35^ which report predicting DXA metrics from 3DO scanning images from 133 participants, where the change in fat mass is slightly underestimated, and lean mass overestimated.

The strengths of our study include the large sample size of the Fenland study, the same DXA instruments in the different samples, the same DXA analytical software, and the robust validation in two separate independent cohorts (Fenland phase 2, representing an older group from the derivation sample and the external validation, which consisted of younger individuals). Furthermore, our method assesses changes to body composition over time. Another benefit of the 3D body mesh approach is to enables anonymity of user data. Our method does not require raw images of the participants to be retained, rather we only store the generated body meshes, which could also be done efficiently by only storing the SMPL shape parameters. This provides additional incentive in scientific and clinical studies for participants to partake in data collection since concern over sharing sensitive information is largely eliminated. Compared to other smartphone apps that estimate body composition from photographs, we note that they either do not reconstruct 3D meshes^21,24^, or they require strict pose constraints^16,24^. Our app works robustly for noisy backgrounds, can be extended to incorporate an arbitrary number of images, and in practice works for arbitrary body and camera poses.

This work is not without limitations. Our optimised body mesh did not achieve a perfect fit to the DXA silhouette. The soft tissues of the DXA participants might be deformed since DXA scans were captured with participants lying flat on the scanbed. This was not modelled by our method. We acknowledge that our study samples were predominantly adults of white European origin. Future analyses should assess the validity of these models in other ethnic groups as well as younger populations since there are significant racial and age differences in body composition^32,36–39^. Future work should also focus on improving the avatar accuracy generated using the smartphone app. While our findings support the validity of our method in Fenland phase 2 data and in the smartphone validation study, we expected and found lower performance using avatars derived from RGB images, as avatars obtained using the phone app can be inaccurate. We do not optimise our smartphone-generated avatars although this would improve prediction accuracy since we do not retain the photographs due to ethical constraints and data security. Alternatively, using domain-agnostic representations such as waist-hip ratio, waist-height ratio might produce stronger results as they are normalised with respect to height. Choudhary et al.^40^ showed that accurate waist-hip ratio could be derived directly from images using attention based networks, which could prove useful in this regard. With improvements to avatar accuracy, app-generated avatars would be able to approach the prediction performance on Fenland.

**Fig. 4:**
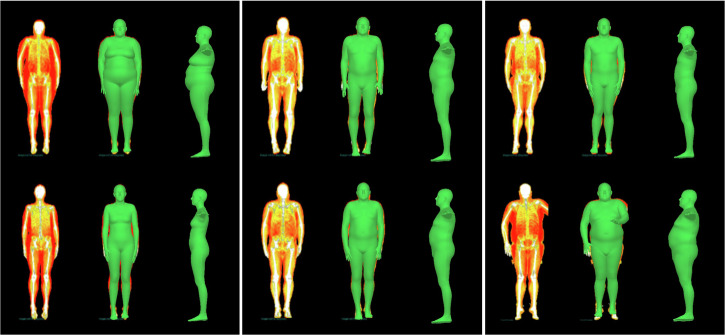
Visualisation of changes to body shape. Each three columns shows one participant who lost (Columns 1–3), maintained (Columns 4–6) or gained (Columns 7–9) fat mass, their DXA scans and fitted meshes for Fenland phase 1 (Row 1) and phase 2 (Row 2). Changes in body shape for the first and third participants are significant.

Through the implementation of the app, users will be able to visualize their body shapes and track potential changes using a portable and relatively inexpensive but accurate device. Using our method in clinical research studies, we could potentially identify individuals at the highest risk of preventable complications (e.g. significant increase in body fat from their first assessment). For instance, Fig. 4 shows the modelled body shapes at phase 1 and 6 years later at phase 2 of three participants who either maintained, gained, or lost fat mass. The first and last three columns show two participants who lost and gained a significant amount of fat mass between the two phases. Significant changes could be seen comparing the two sets of meshes. The end goal would be to encourage users to adopt a healthier way of life, by visualizing changes to their body shapes with time, rather than just focusing on numerical values (e.g. increase in BMI).

In conclusion, capturing 3D body shape using two-dimensional (2D) images coupled with appropriate inference techniques to reconstruct a 3D model of the body, may be a viable alternative tool to clinical medical imaging, and it could offer a readily accessible health metric for monitoring the efficacy of lifestyle interventions.

## Methods

### The Fenland study

DXA scans, paired anthropometry data and metabolic health variables used for our method come from the Fenland study. Details of the study have been described elsewhere^41^. Briefly, the Fenland study is a population-based cohort study established in 2005. It comprises mainly participants of white European descent, born between 1950 and 1975, and recruited from general practice lists in Cambridgeshire (Cambridge, Ely, and Wisbech) in the United Kingdom. A total of 12,435 people took part in Phase 1 of the study (2005–2015) and 7795 in Phase 2 of the study (2014–2020). Exclusion criteria for the Fenland study were pregnancy, diagnosed diabetes, inability to walk unaided, or psychosis. The study was approved by the Cambridge Local Research Ethics Committee and performed in accordance with the Declaration of Helsinki. All participants provided written informed consent to participate in the study.

In the current analyses, we excluded participants whose DXA scans had technical irregularities such as missing tissues or other scan artefacts. The analyses included 11,359 individuals (5333 men and 6026 women) from Phase 1, 6102 individuals from Phase 2 (2979 men and 3123 women), of whom 5733 had valid data from both phases. 80% of the Fenland Phase 1 sample was used for the derivation and training of the 3D body shape composition models, and the remaining 20% of Phase 1 was used to test the validity of those models. The Phase 2 sample was used as a test dataset for validity in a now older population and to assess the sensitivity of prediction models to track within-individual changes over time.

### Smartphone validation study

We also conducted a separate study which in addition to DXA scans also included air plethysmography (BODPOD) and a smartphone app capturing front, back and two-side pose images. This was carried out in a sample of 119 healthy adults (39 men and 80 women) aged between 18 and 65 years old, free from disease and medications between July and November 2023. The participants in this study were recruited locally through advertisements and approval was granted by the Cambridge Central Ethics Committee (REC 06.Q0108.84). Written informed consent was obtained prior to the participants’ visit. This independent sample was used to test the validity of our derived models from the Fenland study, as well as to evaluate the accuracy of the 3D shape obtained from smartphone images alone.

### Anthropometry and body composition

In the Fenland study (both Phase 1 and Phase 2), weight was measured to the nearest 0.2 kg with a calibrated electronic scale (TANITA model BC-418 MA; Tanita, Tokyo, Japan). Height was assessed to the nearest 0.1 cm with a wall-mounted stadiometer (SECA 240; Seca, Birmingham, United Kingdom). Body mass index (BMI; in kg/m^2^) was calculated as weight divided by height squared. Waist circumference and hip circumference were measured to the nearest 0.1cm with a non-stretchable fibre-glass insertion tape (D loop tape; Chasmors Ltd, London, United Kingdom). Waist circumference was defined as the midpoint between the lowest rib margin and the iliac crest, and hip circumference was defined as the widest level over the trochanters. All measurements were taken by trained field workers. Body composition was assessed by DXA, a whole-body, low-intensity X-ray scan that precisely quantifies fat mass in different body regions (models used: Lunar Prodigy Advanced fan beam DXA scanner, or an iDXA system; GE Healthcare, Hatfield, UK). Participants were scanned supine by trained operators, using standard imaging and positioning protocols. All images were manually processed by one trained researcher, who corrected DXA demarcations according to a standardised procedure. In brief, the arm region included the arm and shoulder area (from the crease of the axilla and through the glenohumeral joint). The trunk region included the neck, chest, and abdominal and pelvic areas. The abdominal region (android region) was defined as the area between the ribs and the pelvis and was enclosed by the trunk region. The leg region included all of the area below the lines that form the lower borders of the trunk. The gluteofemoral region (gynoid region) included the hips and upper thighs and overlapped both leg and trunk regions. The upper demarcation of this region was below the top of the iliac crest at a distance of 1.5 times the abdominal height. DXA CoreScan software (GE Healthcare, Hatfield, UK) was used to determine visceral abdominal fat mass within the abdominal/android region. This software uses a proprietary inbuilt algorithm^42^ to derive visceral abdominal fat mass within the android region, validated by the manufacturer using computed tomography and magnetic resonance imaging. The inbuilt algorithm estimates visceral abdominal fat mass by firstly estimating the subcutaneous fat width and the anteroposterior thickness of the abdominal wall. These parameters together with derived geometric constants are implemented to extrapolate the amount of subcutaneous fat mass in the android region. Visceral abdominal fat mass is then calculated by subtracting the estimated subcutaneous abdominal fat mass from the total android fat mass. Subcutaneous abdominal fat is therefore android fat mass minus visceral fat mass. The other body composition variables used in these analysis are derived as follows: Appendicular lean mass (ALM) is the sum of the lean tissue mass in the arms and legs. Appendicular lean mass is scaled to height to derive appendicular lean mass index ALMI (ALM/height^2^)^34^. Peripheral fat mass is the sum of the fat tissue mass in the arms and legs.

In the smartphone validation study, demographic information on age, sex, ethnicity was self-reported. Trained staff acquired all clinical measures. Height and weight were measured using a column scale (Seca GmbH & Co. KG, Hamburg). Waist and hip circumferences were measured using a tape measurer (CEFES-FIBRE by Hoechstmass Germany). Body volume was assessed using air plethysmography (BODPOD ADP system, Cosmed Srl, Rome, Italy), for which participants were in fitted clothing without shoes, and wearing a swim cap before entering the system. Total and regional body composition was measured using an iDXA scanner (GE Healthcare, Hatfield, UK). Four 2D photographs were captured by a smartphone camera (iPhone X, Apple Inc. IOS v15.6.1) using our purpose-built 3D Body Shape app, which constructs a 3D body mesh using phone images only. We adopted a standardised image capture procedure: Participants wore form-fitting clothing, without shoes, and were asked to stand in an ‘A’ pose 2.5 m from the camera; The smartphone was held upright and positioned at chest level; Four photographs consisting of participant front, back, left-side and right-side profiles were taken.

### Model derivation

Firstly, our method fits a 3D body mesh to a DXA silhouette with paired anthropometric measurements (participant height, waist and hip circumferences). Then, the fitted mesh shape parameters (SMPL shape ${\boldsymbol{\beta }}\,\in \,{{\mathbb{R}}}^{10}$) are used to predict body composition metrics. Our smartphone validation study generates 3D body meshes from RGB images by averaging avatars across multiple views according to the uncertainty of shape parameters in each view^31^.

The following describes our fitting pipeline, the body composition regressor, and smartphone avatar generation.

**Fig. 5:**
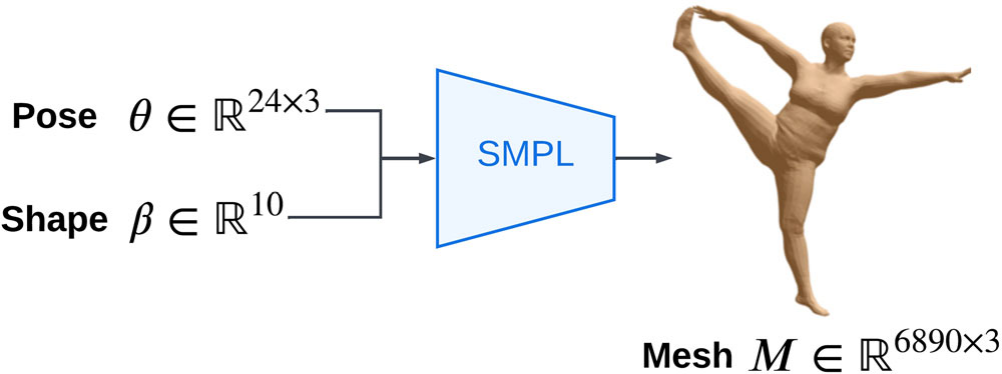
SMPL body model. SMPL model which uses a low-rank PCA representation of the body shape space. The model requires 24 joint rotation parameters and 10 shape parameters as input and returns an expressive body mesh in a differentiable manner. Example avatar is taken from https://meshcapade.wiki/SMPL.

DXA images are single view and orthographic^43,44^, and hence lack depth information. To fit a 3D mesh, we, therefore, augment DXA silhouettes with paired anthropometry measurements, namely height, waist and hip circumferences. Directly fitting a high-dimensional point cloud to a single silhouette is challenging. We utilise Skinned Multi-Person Linear Model (SMPL)^45^, a low-rank PCA shape representation, which provides a strong prior for human body shape. Predicting SMPL pose and shape accurately in one go is also challenging, thus we utilise a two-stage approach where an optimiser refines an initial guess. SMPL^45^ is widely used for Human Pose and Shape (HPS) regression tasks^20,31,46^. Given input pose and shape parameters $\theta \in {{\mathbb{R}}}^{24\times 3},\beta \in {{\mathbb{R}}}^{10}$, SMPL returns a 3D mesh $M\in {{\mathbb{R}}}^{6890\times 3}$ in a fully differentiable manner. Figure 5 shows the structure of the SMPL model.

To reconstruct a 3D mesh from a DXA image, we first make an initial guess of pose and shape using an off-the-shelf method. Most existing HPS networks take in RGB images as inputs and do not readily apply to DXA images^47,48^. Instead, we utilise proxy representations of DXA images, consisting of edge images and joint heatmaps^46^ in an attempt to bridge the domain gap between DXA and RGB images. Our initial pose and shape guess uses Hierarchical Kinematic Probability Distributions (HKPD) by Sengupta et al.^20^, which adopts this proxy representation, and regresses probability distributions over SMPL pose and shape parameters. Regressing distributions also enable us to aggregate information across different views for the smartphone validation study.

While initial predictions from the HKPD method yield an estimation of body pose and shape from a single coronal view of the DXA participant, it is not sufficiently accurate for downstream metric regression tasks. The reason for this is two-fold: firstly, the coronal DXA silhouette provides little information about the body shape in the depth dimension, which is important in terms of assessing body composition metrics such as visceral fat, which is correlated with the sagittal abdominal diameter (SAD)^49^; Secondly, HKPD is trained using synthetic body shapes sampled from Gaussian distributions and tends to predict body shapes biased towards the SMPL mean. Therefore, we construct an optimisation method to refine the initial guess, taking advantage of paired anthropometry measurements, DXA silhouettes, and further losses derived from special properties of DXA scanning (e.g. participants lying flat on the scanbed). We detail the optimisation losses below.

Anthropometry loss. We ensure that the mesh agrees with anthropometry measurements consisting of waist, hip circumferences and height by minimising the following loss:

1 $${{\mathcal{L}}}_{1}={\lambda }_{1}| | {\hat{C}}_{W}-{C}_{W}| {| }_{2}^{2}+{\lambda }_{2}| | {\hat{C}}_{H}-{C}_{H}| {| }_{2}^{2}+{\lambda }_{3}| | \hat{H}-H| {| }_{2}^{2}$$

where $\hat{\cdot }$ are measurements from the optimised mesh. [*C*_*W*_, *C*_*H*_, *H*] stand for waist, hip circumferences and height of the participant respectively. Height of the mesh is measured using the extrema vertices of the T-pose mesh.

To inject waist and hip information to the mesh whilst maintaining differentiability, we use a local ellipsoidal approximation to estimate circumferences. This is done by selecting a ring of key points around waist and hip, then fitting an ellipse using least squares. The selected key points are projected onto the horizontal plane, as SMPL vertices usually do not share the same height, and their relative heights vary across different body shapes.

DXA silhouette loss. Our optimiser also fits to the silhouette of the scan by differentiably rendering the silhouette of the optimised mesh onto the image. We also impose a joint regulariser to prevent the optimised mesh from straying too far from the initial guess. This forms the second part of the loss function,

2 $${{\mathcal{L}}}_{2}={\lambda }_{4}| | {\mathcal{R}}(M(\hat{{\boldsymbol{\theta }}},\hat{{\boldsymbol{\beta }}}),\hat{{\bf{c}}})-{S}_{{\rm{gt}}}\left.\right){\parallel }_{2}^{2}+{\lambda }_{5}\parallel {\hat{J}}_{{\rm{2D}}}-{J}_{{\rm{2D}}}{\parallel }_{2}^{2}$$

where ${\mathcal{R}}(\cdot )$ is a PyTorch3D^50^ differentiable silhouette renderer, **c** = [*s*, *t*_*x*_, *t*_*y*_] are weak perspective camera parameters consisting of scale and translation. *S*_gt_ is the ground truth silhouette obtained by thresholding the DXA image. *J*_2D_ are 2D joint locations obtained by,

3 $${J}_{{\rm{2D}}}=s\Pi ({\mathcal{J}}M(\hat{{\boldsymbol{\theta }}},\hat{{\boldsymbol{\beta }}}))+[{t}_{x},{t}_{y}]$$

where *Π*(⋅) is orthographic projection, ${\mathcal{J}}$ is a linear vertex-to-joint regressor.

Scanbed alignment loss. Due to the orthographic nature of DXA images, pose of the DXA participant can be ambiguous from a single silhouette. As a result, initial guesses from HKPD often produce meshes that have forward-leaning torsos, or legs that are not fully extended. Since DXA participants are lying flat on the scanbed, we impose an additional ‘scanbed alignment constraint’. During implementation, we also impose a pose regulariser on the arms as well as a deviation from z loss to regularise arm poses.

4 $${{\mathcal{L}}}_{3}={\lambda }_{6}| | {\hat{{\boldsymbol{\theta }}}}^{(1)}{| }_{x,y}| {| }_{2}^{2}+{\lambda }_{7}| | {\hat{{\boldsymbol{\theta }}}}^{(2)}-{{\boldsymbol{\theta }}}^{(2)}| {| }_{2}^{2}+{\lambda }_{8}| | {{\bf{z}}}_{{\rm{arms}}}-{z}_{{\rm{pelvis}}}| {| }_{2}^{2}$$

where ***θ***^(1)^ is a subset of SMPL pose parameters containing spine and leg joints, ***θ***^(2)^ is a subset of SMPL pose parameters containing arm and wrist joints.

**Fig. 6:**
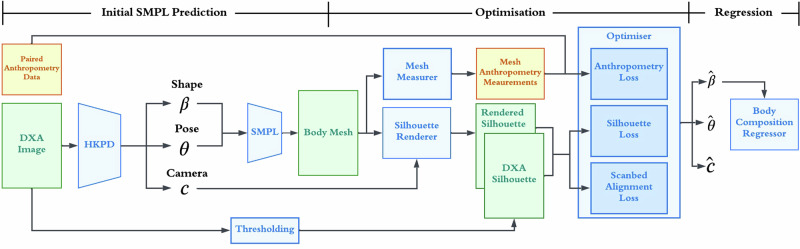
Our 3D body mesh fitting method. Given a DXA image, an initial pose and shape estimation is made using HKPD. Then, the output pose and shape parameters are optimised using losses constructed from DXA silhouettes and paired anthropometry data. Optimised shape parameters are used for body composition regression.

We optimise body pose, shape and camera parameters using the following total loss function,

5 $${\mathcal{L}}({\boldsymbol{\beta }},{\boldsymbol{\theta }},{\bf{c}})={{\mathcal{L}}}_{1}+{{\mathcal{L}}}_{2}+{{\mathcal{L}}}_{3}$$

where *λ*_*i*_ are manually tuned to produce the best fits. Figure 6 shows our full mesh fitting pipeline. Optimised SMPL shape parameters $\hat{{\boldsymbol{\beta }}}\in {{\mathbb{R}}}^{10}$ are used for the next phase which regresses body composition metrics.

**Fig. 7:**
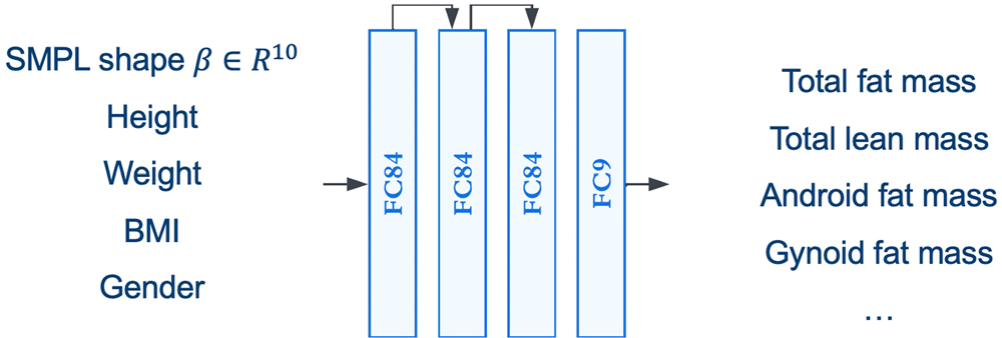
Our body composition regressor. The model is a simple feed-forward neural network which takes in optimised SMPL shape parameters, and outputs mass predictions.

We construct a simple feed-forward neural network to regress body composition metrics from the 3D meshes. The inputs to the network are the 10 SMPL shape parameters obtained from DXA optimisation, height, weight, BMI, and gender of the participant. The outputs of the network are the estimated body composition metrics such as total fat mass, total lean mass, etc. We use residual connections in the first two layers as they slightly improve our lean mass predictions. The structure of our network is shown in Fig. 7.

The network is trained using a mean squared error (MSE) loss function constructed from target and predicted mass values. We weight the loss function using homoscedastic uncertainty^51^, and learn these metric-wise uncertainties automatically,

6 $${\mathcal{L}}({\bf{W}},{\boldsymbol{\sigma }})=\sum _{i}\frac{1}{2{\sigma }_{i}^{2}}| | {\hat{y}}_{i}-{y}_{i}| {| }_{2}^{2}+\mathop{\sum }\limits_{i}\log {\sigma }_{i}$$

where $\hat{\cdot }$ are model predictions, *σ*_*i*_ are metric-wise uncertainties.

The network is relatively small, with around 16,000 parameters, to prevent overfitting. We train the network using an Adam^52^ optimiser for 100 epochs with a learning rate of 0.01. Dropout^53^ is adopted to regularise the network. We train our model using an 80–20% train–validation split on Fenland phase 1 data. We test our method on Fenland phase 2 and the smartphone validation study.

The smartphone validation study uses the HKPD^20^ method and generates SMPL avatars using multiview information from RGB images. Given a group of images [**I**_1_, **I**_2_, …] of the same participant, each photo is firstly processed using HKPD to generate a Gaussian distribution over SMPL shape parameters *p*(***β***∣**I**_*n*_). A final body shape is derived by combining shape information across multiple views according to,

7 $$p({\boldsymbol{\beta }}| {\{{{\bf{I}}}_{n}\}}_{n = 1}^{N})\propto \mathop{\prod }\limits_{n=1}^{N}p({\boldsymbol{\beta }}| {{\bf{I}}}_{n})$$

where we have assumed conditional independence across views^20,31^.

### Statistical analysis

Statistical analyses were performed using STATA version 17 (StataCorp, College Station, Texas, USA) and Python. A *P* value less than 0.05 was considered statistically significant. Descriptive data were reported as mean ± standard deviation (SD) or *n* (%). Using our methods, we constructed our model to predict total and regional body composition metrics. The performance of the derived model was compared by calculating the Pearson correlation coefficients *r* for each outcome parameter and root-mean-square error (RMSE) values. Pearson correlation coefficients were used to investigate associations between the different predicted values of body composition and the measurements of total and regional body composition from DXA. Scatter plots were used to visualise the associations between predicted and measured values. Bland–Altman analysis was used to investigate the agreement between the predicted body composition from our approach against DXA reference measures of total and regional body composition. In the Bland–Altman plot, the y-axis represents the difference or bias between predicted values and measured values (e.g. from DXA) with limits of agreement (LoA) described as the 95% confidence range (mean bias ± 1.96SD), while the x-axis represents the mean value of the reference method (e.g. DXA) rather than the mean between the two methods. Mean differences/biases between the two methods were calculated and significance was tested against zero by paired t-tests. For all the variables, change in body composition was defined as the difference between predictions from Fenland Phase 2 (follow-up assessment) and Fenland Phase 1 (baseline assessment). Scatter plots were used to compare changes in body composition from our predictions with DXA body composition changes. Bland–Altman plots were implemented to assess the agreement between changes in body composition predicted by our method and those measured by DXA. Root-mean-square error (RMSE) was used to assess the accuracy of these comparisons.

## Supplementary information

Supplementary Material

## Data Availability

The datasets generated and analysed during the current study are available at request via the MRC Epidemiology website (http://www.mrc-epid.cam.ac.uk/research/data-sharing/).
