# Supplementary material for: Prediction of total and regional body composition from 3D body shape
Source: NPJ Digit Med. 2024 Oct 23;7:298. doi: 10.1038/s41746-024-01289-0 (PMC11500346; doi:10.1038/s41746-024-01289-0)
Supplement: Supplementary file 1 — Supplementary Material [file 41746_2024_1289_MOESM1_ESM.pdf]

Supplementary Information

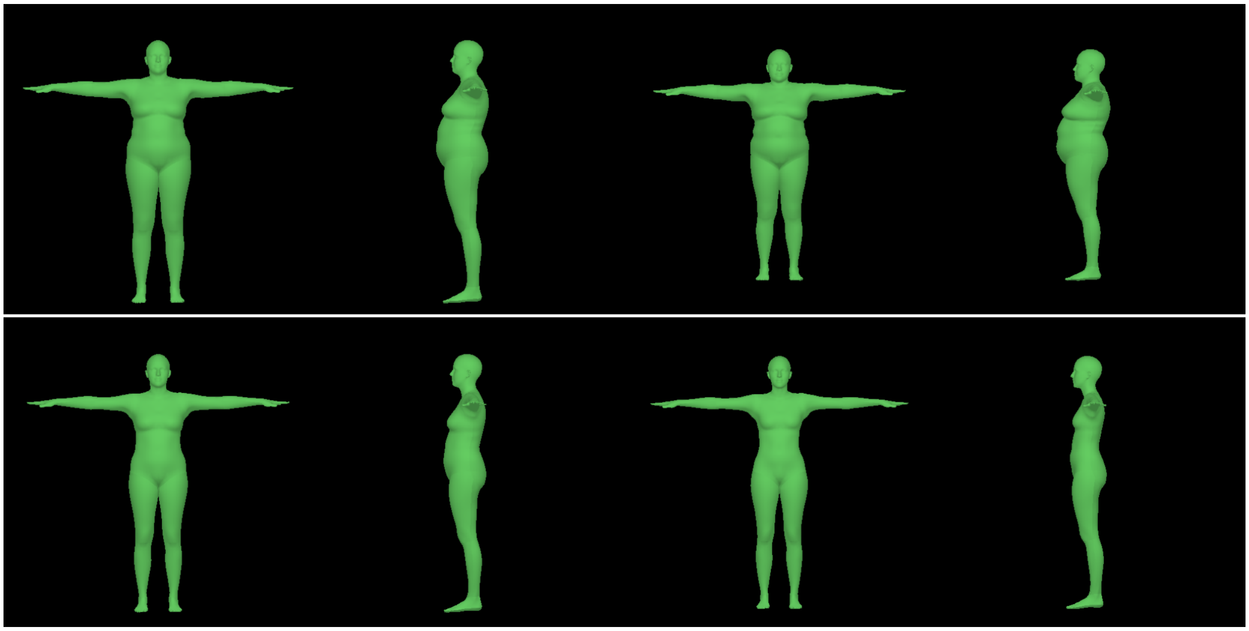

Supplementary Figure 1: Comparison of initial fits and optimised fits. Column 1, 2: Initial body shape fits using HKPD (in T-pose, front and side view); Column 3, 4: Optimised fits (in T-pose, front and side view). Initial fits lack information in the depth axis. Using anthropometry (height, waist, hip circumferences), optimised body meshes reflect the actual body shape much better.

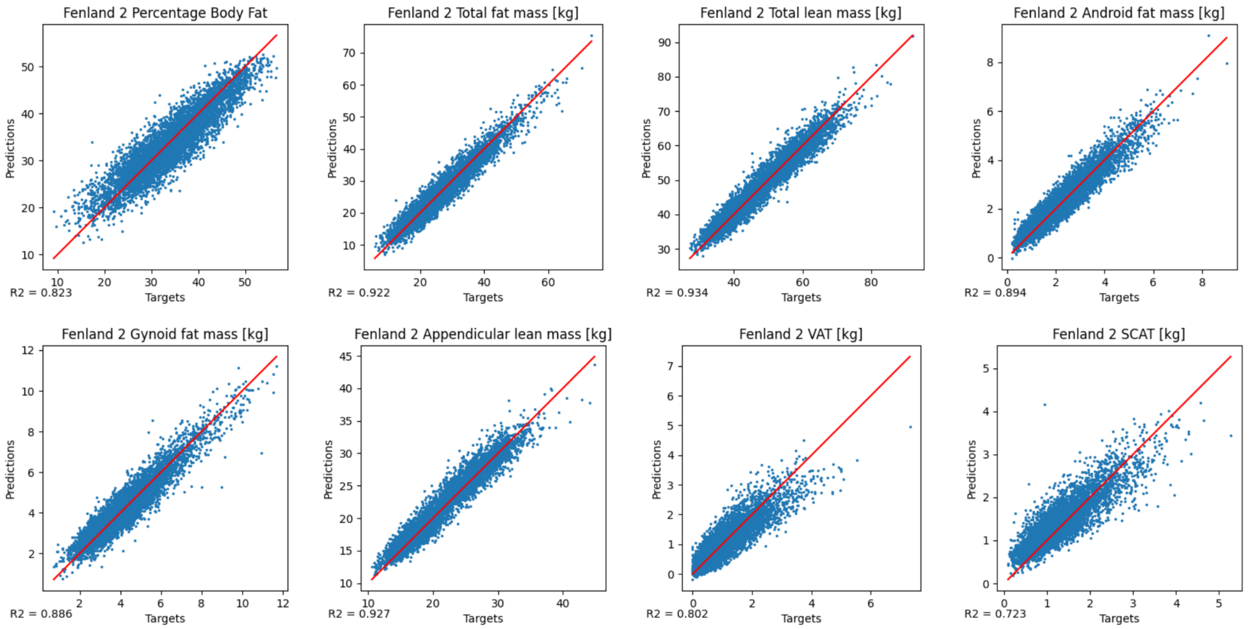

Supplementary Figure 2: Selected scatter plots of model predictions against target values on Fenland 2. Our model is capable of predicting numerous composition metrics with high accuracy.

Supplementary Table 1: Predictions of body fat percentage for the Fenland Phase 2 cohort stratified by BMI and sex.

RMSE [%]	Normal weight	Overweight	Obese	All
Male	3.40	3.03	3.20	3.19
Female	3.71	3.09	2.95	3.38
All	3.59	3.06	3.07	3.28

Categories defined as: Normal weight: BMI $\leq 25kg/m^2$, overweight: $25kg/m^2 <$ BMI $\leq 30kg/m^2$, Obese: BMI $> 30kg/m^2$.
